# Prediction of Recurrent Urinary Tract Infection in Paediatric Patients by Deep Learning Analysis of ^99m^Tc-DMSA Renal Scan

**DOI:** 10.3390/diagnostics12020424

**Published:** 2022-02-06

**Authors:** Hyunjong Lee, Beongwoo Yoo, Minki Baek, Joon Young Choi

**Affiliations:** 1 Department of Nuclear Medicine, Samsung Medical Center, Sungkyunkwan University School of Medicine, Seoul 06351, Korea; nmhjlee@gmail.com; 2 Sungkyunkwan University School of Medicine, Seoul 06351, Korea; ubwoo4175@naver.com; 3 Department of Urology, Samsung Medical Center, Sungkyunkwan University School of Medicine, Seoul 06351, Korea; minki.baek@samsung.com

**Keywords:** deep learning, convolutional neural network, ^99m^Tc-DMSA renal scan, urinary tract infection, prognosis, prediction

## Abstract

*Purpose*: Tc-99m dimercaptosuccinic acid (^99m^Tc-DMSA) renal scan is an important tool for the assessment of childhood urinary tract infection (UTI), vesicoureteral reflux (VUR), and renal scarring. We evaluated whether a deep learning (DL) analysis of ^99m^Tc-DMSA renal scans could predict the recurrence of UTI better than conventional clinical factors. *Methods*: the subjects were 180 paediatric patients diagnosed with UTI, who underwent immediate post-therapeutic ^99m^Tc-DMSA renal scans. The primary outcome was the recurrence of UTI during the follow-up period. For the DL analysis, a convolutional neural network (CNN) model was used. Age, sex, the presence of VUR, the presence of cortical defects on the ^99m^Tc-DMSA renal scan, split renal function (SRF), and DL prediction results were used as independent factors for predicting recurrent UTI. The diagnostic accuracy for predicting recurrent UTI was statistically compared between independent factors. *Results*: The sensitivity, specificity and accuracy for predicting recurrent UTI were 44.4%, 88.9%, and 82.2% by the presence of VUR; 44.4%, 76.5%, and 71.7% by the presence of cortical defect; 74.1%, 80.4%, and 79.4% by SRF (optimal cut-off = 45.93%); and 70.4%, 94.8%, and 91.1% by the DL prediction results. There were no significant differences in sensitivity between all independent factors (*p* > 0.05, for all). The specificity and accuracy of the DL prediction results were significantly higher than those of the other factors. *Conclusion*: DL analysis of ^99m^Tc-DMSA renal scans may be useful for predicting recurrent UTI in paediatric patients. It is an efficient supportive tool to predict poor prognosis without visually demonstrable cortical defects in ^99m^Tc-DMSA renal scans.

## 1. Introduction

Childhood urinary tract infection (UTI) is one of the main bacterial illnesses of concern in paediatrics [1]. Vesicoureteral reflux (VUR) is frequently diagnosed after UTI [2]. Conversely, it is also a main cause of UTI [3]. Both UTI and VUR cause renal scarring or chronic kidney disease (CKD) in severe cases [4,5]. In particular, the nephropathy associated with VUR can manifest decreased renal function with a reduced renal length in renal ultrasonography and a reduced split renal function (SRF) in renal scans [6]. Thus, it is important to predict recurrence of UTI, especially in paediatric patients, in terms of selecting appropriate therapeutic options, determining a follow-up plan, and preventing aggravation of renal dysfunction.

The Tc-99m dimercaptosuccinic acid (^99m^Tc-DMSA) renal scan is a widely utilised nuclear medicine imaging modality; ^99m^Tc-DMSA is a radiopharmaceutical that is absorbed by the renal cortex. When photon defects on the renal cortex are shown in a ^99m^Tc-DMSA renal scan, renal scarring is diagnosed by nuclear medicine physicians. Relative renal function also can be evaluated by comparing the uptake of each kidney. In previous studies, the presence of an abnormality in the ^99m^Tc-DMSA renal scan has been reported as an excellent predictor of recurrent UTI and aggravation of CKD [7,8]. Therefore, ^99m^Tc-DMSA renal scans are performed routinely not only for paediatric patients with initial febrile UTI, but also in the follow-up after a UTI event. However, there are some inconsistencies in which UTI recurs in patients with a normal ^99m^Tc-DMSA renal scan. The inconsistency is readily understandable, as the presence of renal scarring in ^99m^Tc-DMSA renal scans is determined via visual assessment by nuclear medicine physicians.

Deep learning (DL) is an analytic method used to discover the complex structure of large-scale data by using network architecture to transfer information through layers [9]. Recent advances in DL algorithms have greatly influenced the field of medicine, and there is no exception in the analysis of nuclear medicine images [10]. Supervised DL typically predicts a target value of a specific classification or a continuous value with labelled input data. Among supervised DL analysis methods, a convolutional neural network (CNN) model is frequently applied to classify image data, including medical images. The advantages of the CNN model are the relatively low amount of pre-processing, such as automatic feature extraction, and fair classification accuracy compared to other algorithms [11]. Although a previous study attempted DL analysis for ^99m^Tc-DMSA renal scans, there have been no reports on predicting the clinical outcome in paediatric patients with ^99m^Tc-DMSA renal scans [12].

In this study, DL analysis was performed on ^99m^Tc-DMSA renal scans to predict the recurrence of UTI. It was evaluated whether DL predictions based on ^99m^Tc-DMSA renal scans could forecast the recurrence of UTI in paediatric patients better than other conventional clinical factors and image findings.

## 2. Methods

### 2.1. Subjects

**Figure 1:**
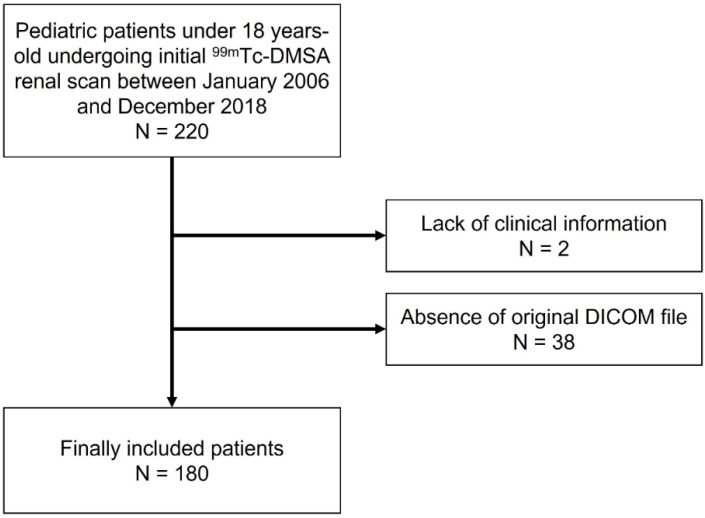
Patient inclusion and exclusion criteria. Two hundred and twenty patients were retrospectively enrolled. Among them, patients with a lack of clinical information or without an original DICOM file were subsequently excluded. Ultimately, 180 patients were included.

Two hundred and twenty consecutive paediatric patients under 18 years old undergoing an initial ^99m^Tc-DMSA renal scan between January 2006 and December 2018 were retrospectively enrolled. The ^99m^Tc-DMSA renal scans were performed after diagnosis of UTI for all patients. Among the sample, two patients were excluded due to a lack of clinical information: one patient with a follow-up duration less than one year and another patient with a lack of SRF data. Another 38 patients were excluded due to the absence of an original DICOM file of the ^99m^Tc-DMSA renal scan. Therefore, 180 patients were ultimately included in this study (Figure 1). Third-generation cephalosporin antibiotic treatment was performed in all patients. In paediatric patients with febrile UTI, a ^99m^Tc-DMSA renal scan is routinely performed for initial workup and follow-up after therapy for evaluating the presence of renal scarring or cortical defects in our institute. All patients underwent a ^99m^Tc-DMSA renal scan within four weeks of antibiotics treatment and clinical follow-up for more than one year. Our institute review board approved this retrospective cohort study (IRB #2020-06-116), and the informed consent requirement was waived.

### 2.2. ^99m^Tc-DMSA Scan Acquisition and Pre-Processing

The ^99m^Tc-DMSA renal scans were performed according to the guidelines of the Society of Nuclear Medicine. Briefly, 37–185 MBq of ^99m^Tc-DMSA was injected into the patients. Image acquisition was performed three hours after the intravenous injection of ^99m^Tc-DMSA by using three dedicated dual-headed gamma cameras (E.CAM, Siemens Healthineers, Erlangen, Germany: pixel size of 1.6 × 1.6 mm; INFINIA, GE Healthcare, Milwaukee, WI, USA: pixel size of 1.4 × 1.4 mm; Discovery NM830, GE Healthcare: pixel size of 2.2 × 2.2 mm). Images were acquired in the posterior, both posterior oblique projections, and both anterior oblique projections in supine position using a dedicated gamma camera. The posterior planar images were used to evaluate SRF. Regions of interest were drawn manually along the margin of each kidney. The count ratio of each kidney was defined as SRF. All images were reviewed by two board-certified nuclear medicine physicians with over five years of experience. The focal prominent photon defect in the renal cortex was considered as a cortical defect. For image reading by nuclear medicine physicians, all projection images were used.

The ^99m^Tc-DMSA renal scan images were obtained as original DICOM files of posterior images (256 × 256 pixels) from the Picture Archiving and Communication Systems department of our institute. Subsequently, any identifying personal information was removed from the header of images. As pre-processing for DL analysis of ^99m^Tc-DMSA renal scan images, the kidney with smaller uptake (lower SRF) was cropped to 64 × 64 pixels manually. To enhance uniformity of the dataset and increase efficacy of DL, images of the left kidney were inverted into right. Image pre-processing was conducted using numPy library version 1.21.0 in Python.

### 2.3. Convolutional Neural Network

CNN was selected as an appropriate analytic tool in this study. It is a supervised DL method to employ mathematical operation between matrixes called convolution [11]. In particular, it has good performance in learning and classification of images. A CNN model was constructed for DL analysis of the ^99m^Tc-DMSA renal scans. Manually cropped ^99m^Tc-DMSA renal scan images were fed into the 2D-CNN model. Three sequential convolution layers, a rectified linear unit, and pooling layers were applied, and the features were fed into a fully connected layer. Dropout layers were applied after the fully connected layers. The outputs of the network were probabilistic scores for the presence of recurrent UTI with values that ranged from zero to one. The final output of CNN was a series of discrete values for prediction of recurrent UTI.

To assess model generalizability and select appropriate hyperparameters, we used a stratified k-fold cross-validation approach three times. In each cross-validation fold, the CNN was trained on 2/3 of the samples and validated on an unseen subset of 1/3 of the samples. This was repeated until each fold had served as the test set. Based on the average accuracy of cross-validation, the hyperparameters of the model were optimized. The sizes of the convolution filters were (3 × 3), (3 × 3), and (3 × 3) in the sequences. The size of the pooling layers was (2 × 2), and after each convolutional layer, batch normalization was applied. The size of the final linear layer was 23. The learning was performed for 30 epochs. The construction and learning of the CNN model were conducted using pytorch library version 1.10 in Python.

### 2.4. Clinical Variables and Statistical Analyses

The primary outcome was the recurrence of UTI during clinical follow-up. Age, sex, presence of VUR, presence of cortical defect on ^99m^Tc-DMSA renal scan, SRF, and DL prediction results were used as independent variables for predicting recurrent UTI. Clinical data including demographics, performance of treatment, and the presence of recurrent UTI were collected retrospectively from the Electronic Medical Records.

Diagnostic efficacy for predicting recurrent UTI was calculated for each clinical factor, image finding, and the results of the CNN classification. The optimal cut-off of SRF was defined as the value that produced the largest area under the receiver operating characteristic (ROC) curve to predict recurrent UTI. Patients with lower SRF than an optimal cut-off value were classified as patients with low SRF. Sensitivity, specificity, and diagnostic accuracy were used to demonstrate diagnostic efficacy. Those of other variables were statistically compared with those of CNN prediction results using the McNemar test. A *p*-value lower than 0.05 was considered statistically significant. All the analyses were performed using Python version 3.7.0.

## 3. Results

### 3.1. Demographic Data

During clinical follow-up, recurrent UTI occurred in 27 of 180 patients (one-year recurrence rate: 15.0%). The median time of the follow-up was 2118 days, and the interquartile range was 2777 days. There were no significant differences in age and sex between patients with and without recurrent UTI. In contrast, significant differences were found in the presence of VUR, presence of cortical defect, and low SRF on ^99m^Tc-DMSA renal scans between those two groups. Detailed demographic data are provided in Table 1.

### 3.2. Diagnostic Accuracy

**Figure 2:**
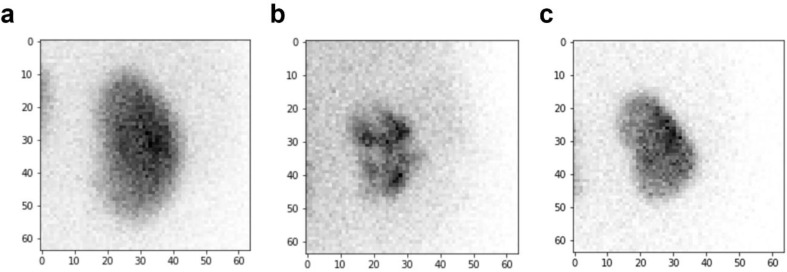
Representative cases of ^99m^Tc-DMSA renal scan. (**a**) A case without demonstrable abnormality in ^99m^Tc-DMSA renal scan and without recurrent UTI. It was classified as a case without recurrent UTI by CNN prediction. (**b**) A case with multiple cortical defects in ^99m^Tc-DMSA renal scan and with recurrent UTI. It was classified as a case with recurrent UTI by CNN prediction. (**c**) A case without demonstrable abnormality in ^99m^Tc-DMSA renal scan and with recurrent UTI. It was classified as a case with recurrent UTI by CNN prediction. ^99m^Tc-DMSA, Tc-99m dimercaptosuccinic acid; UTI, urinary tract infection; CNN, convolutional neural network.

The optimal cut-off of SRF to predict recurrent UTI was set as 45.9% based on ROC curve analysis. The area under ROC curve was 0.816 (Supplementary Figure S1). The sensitivity, specificity and accuracy for predicting recurrent UTI were 44.4%, 88.9%, and 82.2% by the presence of VUR; 44.4%, 76.5%, and 71.7% by the presence of cortical defect; 74.1%, 80.4%, and 79.4% by low SRF; and 70.4%, 94.8%, and 91.1% by the CNN prediction results. There were no significant differences in sensitivity of the CNN prediction results compared to the other variables. However, the specificity or accuracy of the CNN prediction results were significantly higher than those of the other variables (Table 2). Representative cases are displayed in Figure 2.

## 4. Discussion

In this study, DL-based analysis of ^99m^Tc-DMSA renal scans was performed to predict recurrence of UTI in paediatric patients. It demonstrated better ability to predict recurrent UTI compared to conventional clinical methods. Few studies have analysed ^99m^Tc-DMSA renal scan images using artificial intelligence algorithms. Lin et al. revealed that artificial intelligence algorithms support reducing the scan time of ^99m^Tc-DMSA single-photon emission computed tomography (SPECT) [13]. However, this study only focused on diagnostic accuracy for the presence of cortical defects, not on clinical outcomes. Wright et al. performed classification of ^99m^Tc-DMSA renal scan images using artificial neural networks [12]. As in the previously cited study, abnormality was set as the binary endpoint of their study. Compared to previous studies, an advantage of the present study is that the endpoint was the presence of recurrent UTI, not abnormality of the image. This is the first study to apply a CNN for ^99m^Tc-DMSA renal images to predict the clinical outcome.

There is a limitation to assessing the presence of cortical defects by visual assessment due to the low resolution of planar ^99m^Tc-DMSA renal scans. In addition, there is inter-observer variability in detecting photon defects between nuclear medicine physicians [14]. Therefore, a previous study evaluated cortical defects using ^99m^Tc-DMSA renal SPECT/Computed Tomography (CT) to enhance the diagnostic power of ^99m^Tc-DMSA renal scans. SPECT/CT showed better detection power for cortical defects compared to planar ^99m^Tc-DMSA renal scanning [15]. Although many scholars have reported the diagnostic accuracy of ^99m^Tc-DMSA renal scans, there is an intrinsic limitation in the concept of ‘diagnostic accuracy.’ It is difficult to define the gold standard for the presence of cortical defects, as pathological evidence is rarely obtained to diagnose cortical defects due to invasiveness and low cost-effectiveness. Although kidney ultrasonography is a frequently used reference modality, it also has limitations of low resolution and indirect diagnosis as an imaging study. From a clinical perspective, diagnosis of cortical defect is meaningful when it shows a significant ability to predict kidney function decrease or the presence of recurrent UTI regardless of its consistency with the presence of pathological cortical defects. Therefore, this study has better clinical impact as the endpoint is not abnormality of image, but prognosis.

It is important to identify patients with the potential for recurrent UTI in terms of follow-up and treatment. Different follow-up intervals may be recommended according to the risk of recurrent UTI stratified by grade of VUR [3]. The presence of bowel/bladder dysfunction and of renal scarring were indicated as other risk factors of recurrent UTI [16]. From a treatment perspective, long-term and low-dose antibiotic use was revealed to reduce the risk of recurrent UTI in predisposed paediatric patients [17]. Based on this study, we suggest that the prediction of recurrent UTI by CNN analysis of ^99m^Tc-DMSA renal scans can be useful with high-risk children, as the diagnostic accuracy was superior to that of conventional clinical methods and image finding. It is recommended that frequent follow-up and long-term low-dose antibiotic prophylaxis be considered for patients with ^99m^Tc-DMSA renal scans that were classified as having a high probability of recurrent UTI. Further clinical study can be conducted to evaluate usefulness of DL analysis in ^99m^Tc-DMSA renal scans to select high-risk paediatric patients for close follow-up and antibiotic prophylaxis.

Recently, image classification using artificial intelligence has been applied widely in various fields, including nuclear medicine imaging [10]. Among numerous analysis methods, CNN is one of the simplest and most powerful tools for image classification [18]. For clinical applications, a reference dataset and pre-learned neural network are needed to classify a new case. In this study, we could retrospectively recruit only 180 patients satisfying the inclusion criteria, even though patients with ^99m^Tc-DMSA renal scans from 2006–2018 were explored. Therefore, it was difficult to propose a complete reference dataset and pre-learned model for clinical use. Further study is planned to recruit more patients continuously, not only from our institute but also from other institutes with multicentre settings.

There were several limitations in this study. Firstly, there were only 27 patients (15.0%) with an event (recurrent UTI) among the 180 total included patients. Therefore, it was difficult to prepare an additional test set for internal validation. To overcome this limitation, stratified k-fold cross-validation was performed. However, further study is needed to substantiate the high diagnostic accuracy of CNN in ^99m^Tc-DMSA renal scanning with larger amounts of internal and external validation data. Secondly, only supervised learning was applied in this study. As supervised learning used labelled data, diagnostic accuracy may be over-estimated in the identical dataset. Unsupervised learning methods such as an autoencoder have been used recently to analyse medical images. It is expected that further study with unsupervised learning would support the results of this study. Finally, ^99m^Tc-DMSA renal scan images were acquired by multiple instruments. Although the technical parameters of each instrument are different, DL analyses are not based on the quantitative value of each pixel but on inter-pixel or inter-region relationship patterns. Therefore, the analysis for integrated data is reasonable.

Taken together, the present study showed that prediction results of CNN in ^99m^Tc-DMSA renal scan had significantly higher specificity and accuracy to diagnose recurrent UTI compared to other conventional clinical factors or image findings. DL analysis of ^99m^Tc-DMSA renal scans may be useful for predicting recurrent UTI in paediatric patients.

## Figures and Tables

**Table 1:** Clinical characteristics of patients.

Characteristics.	Overall	Presence of Recurrent UTI	No Recurrent UTI	*p*
	(*n* = 180)	(*n* = 27)	(*n* = 153)	
Age (range, years)	1.6 (0.1–17.6)	1.5 (0.1–9.8)	1.7 (0.1–17.6)	0.368
Sex, male	109 (60.6%)	17 (63.0%)	92 (60.1%)	0.781
Presence of VUR	29 (16.1%)	12 (44.4%)	17 (11.1%)	< 0.001
Presence of cortical defect	48 (26.7%)	12 (44.4%)	36 (23.5%)	0.023
SRF (range, %)	46.0 (7.3–50.0)	39.4 (7.3–49.4)	46.9 (15.5–50.0)	< 0.001

Note: Data are numbers of patients (proportion). UTI, urinary tract infection; VUR, vesicoureteral reflux; SRF, split renal function.

**Table 2:** Diagnostic efficiency for recurrent UTI in each independent variable.

.	Sensitivity	*p*	Specificity	*p*	Accuracy	*p*	PPV	NPV	PLR	NLR
Presence of VUR	44.4% (12/27)	0.211	88.9% (136/153)	0.110	82.2% (148/180)	0.030	41.4% (12/29)	90.1% (136/151)	4	0.63
Presence of cortical defect	44.4% (12/27)	0.211	76.5% (117/153)	<0.001	71.7% (129/180)	<0.001	25% (12/48)	88.6% (117/132)	1.89	0.73
Low SRF	74.1% (20/27)	0.999	80.4% (123/153)	0.001	79.4% (143/180)	0.006	40% (20/50)	94.6% (123/130)	3.78	0.32
CNN prediction result	70.4% (19/27)		94.8% (145/153)		91.1% (164/180)		70.4% (19/27)	70.4% (19/27)	13.54	0.31

Note: *p*-values represent statistical significances of McNemar test between each independent variable and CNN prediction result. VUR, vesicoureteral reflux; SRF, split renal function; CNN, convolutional neural network, PPV, positive predictive value; NPV, negative predictive value; PLR, positive likelihood ratio; NLR, negative likelihood ratio.

## Data Availability

The data that support the findings of this study are available from the corresponding author, upon reasonable request.

## Supplementary Materials

The supporting information can be downloaded at: https://www.mdpi.com/article/10.3390/diagnostics12020424/s1, Figure S1: ROC curve for selecting optimal cut-off of SRF.
