# Supplementary material for: Prediction of Recurrent Urinary Tract Infection in Paediatric Patients by Deep Learning Analysis of 99mTc-DMSA Renal Scan
Source: Diagnostics (Basel). 2022 Feb 6;12(2):424. doi: 10.3390/diagnostics12020424 (PMC8870906; doi:10.3390/diagnostics12020424)
Supplement: Supplementary file 1 [file diagnostics-12-00424-s001.zip › diagnostics-1563078-supplementary.pdf]

SUPPLEMENTARY FIGURES

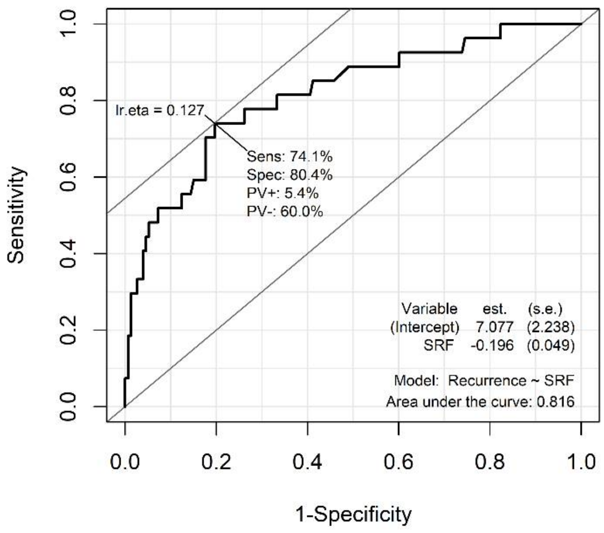

Supplementary Figure S1. ROC curve for selecting optimal cut-off of SRF.

The optimal cut-off of SRF to predict recurrent UTI was set as 45.9% based on ROC curve analysis.
ROC, receiver operating characteristic; SRF, split renal function
